# Mitigating effects of L-selenomethionine on low-dose iron ion radiation-induced changes in gene expression associated with cellular stress

**DOI:** 10.3892/ol.2013.1362

**Published:** 2013-05-23

**Authors:** MANUNYA NUTH, ANN R. KENNEDY

**Affiliations:** Department of Radiation Oncology, University of Pennsylvania School of Medicine, Philadelphia, PA 19104, USA

**Keywords:** ionizing radiation, HZE particles, L-selenomethionine, microarray

## Abstract

Ionizing radiation associated with highly energetic and charged heavy (HZE) particles poses a danger to astronauts during space travel. The aim of the present study was to evaluate the patterns of gene expression associated with cellular exposure to low-dose iron ion irradiation, in the presence and absence of L-selenomethionine (SeM). Human thyroid epithelial cells (HTori-3) were exposed to low-dose iron ion (1 GeV/n) irradiation at 10 or 20 cGy with or without SeM pretreatment. The cells were harvested 6 and 16 h post-irradiation and analyzed by the Affymetrix U133Av2 gene chip arrays. Genes exhibiting a 1.5-fold expression cut-off and 5% false discovery rate (FDR) were considered statistically significant and subsequently analyzed using the Database for Annotation, Visualization and Integrated Discovery (DAVID) for pathway analysis. Representative genes were further validated by real-time RT-PCR. Even at low doses of radiation from iron ions, global genome profiling of the irradiated cells revealed the upregulation of genes associated with the activation of stress-related signaling pathways (ubiquitin-mediated proteolysis, p53 signaling, cell cycle and apoptosis), which occurred in a dose-dependent manner. A 24-h pretreatment with SeM was shown to reduce the radiation effects by mitigating stress-related signaling pathways and downregulating certain genes associated with cell adhesion. The mechanism by which SeM prevents radiation-induced transformation *in vitro* may involve the suppression of the expression of genes associated with stress-related signaling and certain cell adhesion events.

## Introduction

Exposure to ionizing radiation poses a threat to astronauts on space missions. Of particular concern is the radiation emitted from high atomic mass (Z) and high-energy (HZE) particles. Unlike the relatively infrequent energy deposition by low linear energy transfer (LET) radiation along its track, high LET radiation from HZE particles exhibits a highly energetic and dense core and a laterally extending secondary radiation. The secondary radiation, termed δ-rays, generates an external region called the penumbra as a consequence of the HZE particle traversing through a medium. It has been estimated that there are approximately 32 cells hit by δ-rays for each cell traversed by a primary HZE particle of 1 GeV/n iron ions (1). The combination of the highly energetic core and δ-rays suggests a more damaging effect of HZE particle radiation compared to that observed from the same dose of low LET radiation. For example, the ratio of double-strand breaks to single-strand breaks is observed to be higher for HZE particle radiation when compared to photon radiation (2). There is evidence that differentially expressed genes respond uniquely to either photons or HZE particles (3). The number and types of gene expression changes attributable to δ-rays are thought to be similar to those expected from exposure to other types of low LET radiation (4). In addition, a significant number of genes not associated with DNA damage have been shown to be responsive to ionizing radiation (5). The response of target genes for products necessary for cell communication, such as those involved with the extracellular matrix (ECM) (6) and gap junctions (e.g., connexin 43) (7), further suggests an important role of ionizing radiation in altering events other than those associated with the DNA damage/repair response.

Ionizing radiation is typically viewed as a genotoxic stress to cells. As such, growth arrest of radiation-damaged cells through the control of cell cycle checkpoints allows the cells to recognize and repair DNA and other damage before DNA synthesis or mitosis occurs (8), whereas apoptosis serves as the means for removing cells with irreparable damage from the cell population. Studies of the effects of low doses have been difficult due to the inherently small changes in gene expression patterns. However, experiments with X- and γ-rays at doses ranging from 0.02 to 20 Gy have previously been successful in demonstrating the effects of low LET radiation on gene expression by microarray analyses (9–12). Low-dose experiments (0.2–2 Gy γ-rays) on peripheral blood cells revealed the induction of genes up to 72 h post-irradiation following 0.2-Gy exposure, whereby a linear dose-response correlation was observed between 0.2 and 2 Gy over the 24-48 h post-irradiation period for selected genes associated with growth arrest, such as CDKN1A/WAF1 and GADD45A (10). Exposures of human fibroblasts to 0.02 and 4 Gy X-rays over a time course of 1–24 h revealed the expression of genes exclusive to low or high-dose exposures, with overlaps (4). While the alteration of gene expression for genes associated with cell-cell signaling and DNA damage response was observed following exposure to a low dose, apoptosis and proliferation genes were modulated following exposure to a high dose of radiation (11).

Selenium is known to be a cancer chemopreventive agent (13) with anticarcinogenic activities against the development of cancers occurring in several different organ systems, such as the prostate, lung and colon (14,15). Selenite and L-selenomethionine (SeM) are the two forms of seleno compounds used in most cancer prevention studies. Although the mechanism for their mode of action is not clear, their ability to promote apoptosis (15) and cytotoxicity (16) in various cancer cell lines suggests a pro-oxidant effect from the inorganic form of selenium. SeM is the organic form of selenium found in selenized yeast supplements and used in clinical trials. The ability of SeM to effectively mitigate oxidative stress *in vitro* and *in vivo* (17–19) supports a role for SeM in antioxidant activities. Moreover, SeM treatment was shown to suppress iron ion radiation-induced transformation in human thyroid epithelial cells (HTori-3) (17).

It was reported previously that a 10 cGy dose to HTori-3 cells did not affect cell survival levels, and that a 20 cGy dose led to cell survival levels of approximately 85% in cells exposed to iron ion irradiation (20). In the current study, genomic profiling was performed to assess the effects of non-toxic (10 cGy) and slightly toxic (20 cGy) radiation exposure in cultured HTori-3 cells in the presence and absence of SeM.

## Materials and methods

### Cell culture and radiation exposure

Human thyroid epithelial cells (HTori-3) were maintained in Dulbecco’s modified Eagle’s medium (DMEM)/F12 supplemented with 1% glutamine and 10% FBS (growth medium). Twenty-four hours prior to irradiation with iron ions, fresh medium with or without 5 *μ*M SeM (Sigma-Aldrich, St. Louis, MO, USA) was added. At the time of radiation exposure, the cells were approximately 80% confluent. Irradiation was performed at the NASA Space Radiation Laboratory (NSRL) facility at the Brookhaven National Laboratory (Upton, NY, USA). Radiation exposure was from 1 GeV/n iron ions delivered as a horizontal beam of approximately 20×20 cm in dimension at a dose rate of approximately 40 cGy/min. Six or 16 h post-irradiation, the cells were harvested and frozen in RNAlater solution (Qiagen, Valencia, CA, USA). Three replicates of two independent experiments were generated for each radiation dose/SeM supplement combination. For sham-irradiated controls, SeM treated or untreated cells were maintained in the same manner as utilized for the irradiated cells at the NSRL facility. For mock SeM pretreatment, the medium was supplemented with phosphate-buffered saline (PBS).

### RNA preparation, microarray and real-time RT-PCR

RNA was extracted from frozen cells using the RNeasy kit (Qiagen) according to the manufacturer’s instructions. Each microarray probe was prepared and hybridized at the Penn Bioinformatics Core (University of Pennsylvania) using 1 *μ*g total RNA. First-strand cDNA was synthesized using Superscript II First Strand cDNA Synthesis System (Invitrogen, Carlsbad, CA, USA). Following RNA degradation with RNase H, second-strand cDNA was synthesized with DNA polymerase I and extracted with 25:24:1 (v/v) phenol:chloroform:isoamyl alcohol. The double-stranded cDNA was used as a template to generate biotinylated cRNA using the BioArray HighYield RNA Transcript Labeling kit (Enzo Life Sciences, Farmingdale, NY, USA). The resulting cRNA was purified, fragmented and hybridized to U133Av2 Gene Chips (Affymetrix, Santa Clara, CA, USA) according to the manufacturer’s instructions, and further processed at the Penn Bioinformatics Core.

For real-time RT-PCR analysis, cDNA was initially synthesized with Superscript II using ∼1-2 *μ*g total RNA. Two-step PCR (initial denaturation, 95°C, 30 sec; 40–50 cycles of 95°C, 5 sec and 65°C, 34 sec) was monitored in real-time by the SYBR-Green DNA intercalating dye (SYBR Advantage qPCR Premix; Clontech Laboratories, Inc., Mountain View, CA, USA) according to the manufacturer’s instructions on an Applied Biosystems 7300 Real-Time PCR System instrument. Primers were designed by ProbeFinder (Roche Applied Science, Indianapolis, IN, USA) or Primer Designer (equipped with the LightCycler real-time RT-PCR instrument, Roche Applied Science) with both β-actin and GAPDH serving as reference genes. The primer sequences used are shown in Table I. PCR products were confirmed by melting curve analysis and/or running on 1% agarose gels. cDNA microarray analysis was performed with biological triplicates, whereas n=4–6 for real-time RT-PCR analyses.

### Statistical analysis and DAVID

Probe intensities were summarized and normalized using log scale robust multi-array analysis (RMA). ANOVA was performed using the GeneSpring GX software (Agilent, Santa Clara, CA, USA) using a 1.5-fold minimum expression cut-off and a false discovery rate (FDR) of 5%. Determination of differential gene expression was performed using dose (sham irradiation, 10 or 20 cGy exposure), time (6 or 16 h post-irradiation) or SeM treatment as independent variables. The Database for Annotation, Visualization and Integrated Discovery (DAVID) (21) was used for functional annotation and analysis by uploading Affymetrix-based nomenclature of statistically significant genes (1.5-fold expression cut-off; 5% FDR; P<0.05; http://david.abcc.ncifcrf.gov). Gene enrichment for pathway analysis (KEGG pathway) was accomplished by the integrated Expression Analysis System Explorer (EASE), with a count threshold of 2 (minimum number of genes for a particular term) and EASE threshold of 0.1 (a modified Fisher’s exact P-value). The Student’s t-test was used for statistical comparisons of real-time RT-PCR results.

## Results

### Global gene expression change in response to low-dose iron ion irradiation

**Figure 1:**
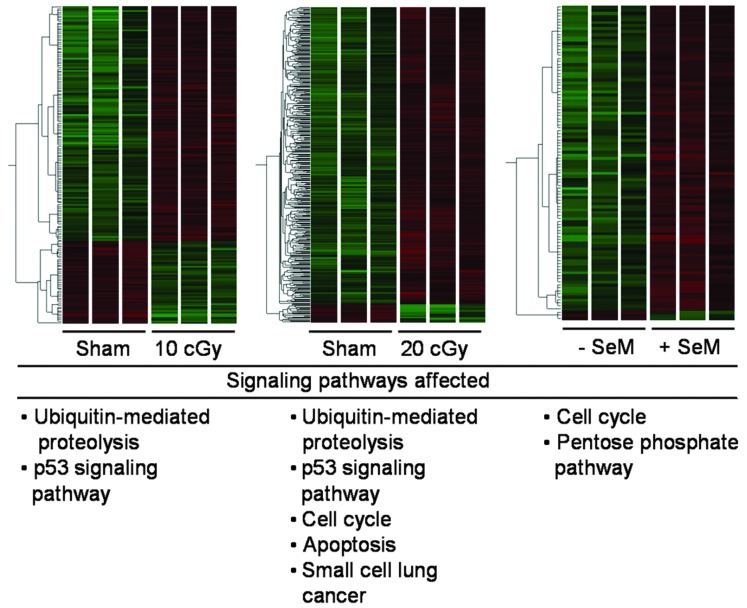
Gene expression profiles of human thyroid epithelial cells (HTori-3) in response to low-dose iron ion irradiation and the associated pathways affected. Cell monolayers were exposed to sham radiation or iron ion radiation (10 or 20 cGy) and processed 6 h after exposure. For L-selenomethionine (SeM) treatment, cell monolayers were sham-treated or treated with 5 *μ*M SeM for 24 h prior to irradiation and similarly processed. The heatmaps depict hierarchical expression clustering, where genes are depicted as upregulated (red) or downregulated (green) using the GeneSpring GX software. Biological triplicates were used for ANOVA analyses, with depicted genes having 1.5-fold gene expression cut-off levels and 5% false discovery rate (FDR). KEGG pathway analysis was performed by the Expression Analysis System Explorer (EASE) algorithm integrated into the Database for Annotation, Visualization and Integrated Discovery (DAVID), setting count threshold=2 and EASE threshold=0.1. The listed pathways are affected by upregulated genes, whereas no pathways were significantly affected by the downregulated genes. A total of 163 and 319 unique genes were identified as responsive to irradiation following removal of redundant terms by DAVID for 10 and 20 cGy doses, respectively.

To determine the impact of low-dose HZE particle radiation on gene expression, HTori-3 cells were exposed to 10 or 20 cGy iron ion irradiation (in the absence of SeM) and compared to the sham-irradiated cells at 6 h post-irradiation. With the GeneSpring software, global gene expression patterns are illustrated as heatmaps depicting the clustering of gene expression upon exposure to radiation (Fig. 1). Pooled averages from three independent experiments were compared. At a dose of 10 cGy, 121 and 42 unique genes were shown to be up- and downregulated, respectively, following the removal of redundant terms by DAVID. Pathway analysis by the KEGG pathway revealed the upregulation of genes associated with ubiquitin-mediated proteolysis and the p53 signaling pathway (Fig. 1 and Table II). Following an increased exposure to the 20 cGy dose, 285 unique genes were upregulated, while 34 genes were downregulated. Moreover, genes associated with the cell cycle, apoptosis and small cell lung cancer were observed to be upregulated, in addition to the pathways elicited at 10 cGy (Fig. 1 and Table III). No significant pathways were identified by DAVID for down-regulated genes in either the 10 or 20 cGy dose group. As such, the expression of genes consistent with a stress response was observed even at non-toxic doses. In addition, the dose increase (from 10 to 20 cGy) contributed to more stress. Lists of the total number of differentially expressed genes are provided in Tables II and III.

### SeM pretreatment alone promotes the upregulation of genes

HTori-3 cells were pretreated with 5 *μ*M SeM for 24 h, processed 6 h post-sham irradiation, and compared to the control cells (mock SeM pretreatment and sham irradiation). In comparison to the untreated cells, 100 unique transcripts were differentially modulated, with 97 and 3 genes up- or down-regulated (Fig. 1 and Table IV). Pathway analysis revealed that the upregulation of genes was associated with the cell cycle and pentose phosphate pathway, with no identifiable pathways affected by the downregulated genes.

### SeM supplementation mitigates gene expression associated with the cellular stress response from 10 cGy irradiation

**Figure 2:**
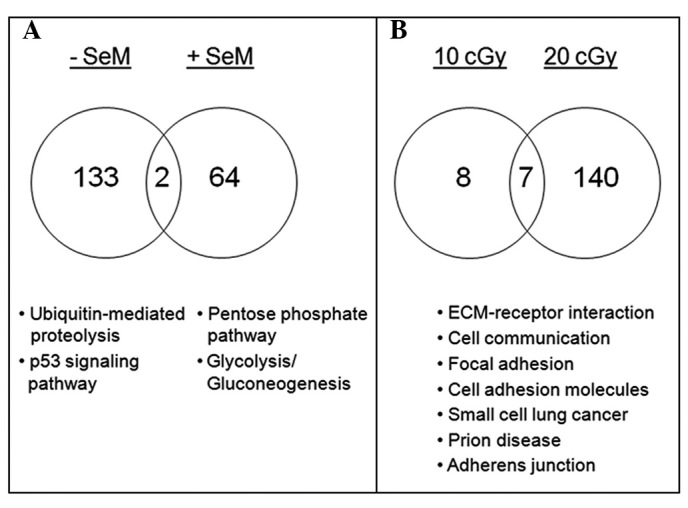
Venn diagrams comparing the effects of SeM treatment on gene expression. (A) Comparison of upregulated genes in cells exposed to a 10 cGy dose in the absence and presence of SeM and processed 6 h after exposure. Corresponding genes and fold induction are shown in Table II. (B) Comparison of total genes affected by SeM pretreatment at varying radiation doses and processed 16 h after exposure. At 20 cGy, genes associated with cell communication are observed to be downregulated and are shown in Table V. Corresponding pathways affected by the treatments are listed below each diagram for both panels. The numbers of altered genes common to treatment comparisons are shown but did not significantly contribute to functional analysis. SeM, L-selenomethionine.

**Figure 3:**
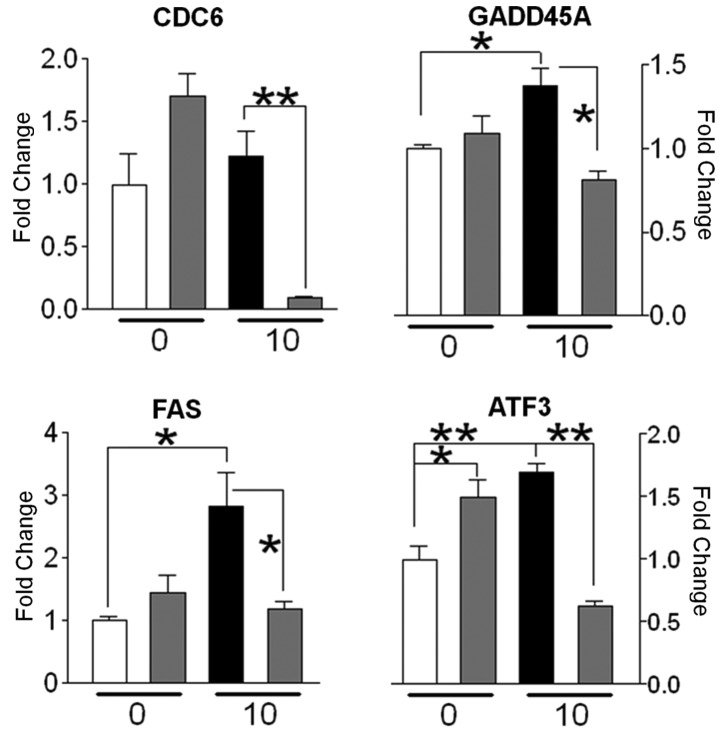
Real-time RT-PCR results of putative genes associated with stress response. Relative expression (fold change) of mRNA transcripts are shown for sham-irradiated controls (clear), SeM-pretreated (gray) and irradiated, mock SeM-pretreated (black) cells. Representative radiation doses are shown for each group (0 and 10 cGy). ^*^P< 0.05 and ^**^P<0.001. Values are means ± SEM for n=4–6. SeM, L-selenomethionine.

As shown in Fig. 2A, SeM supplementation in irradiated cell cultures resulted in a reduction in the number of regulated genes representing less than half of the total differentially expressed transcripts, in comparison to the untreated population. Cells were irradiated with 10 cGy iron ions in the absence and presence of SeM pretreatment and processed 6 h post-irradiation. Datasets from both radiation treatments were initially compared pairwise to the sham-irradiated samples and then compared for SeM treatment. Upon SeM pretreatment, ubiquitin-mediated proteolysis and p53 signaling (Table II) were no longer significant. Moreover, genes associated with the pentose phosphate pathway and glycolysis/gluconeogenesis were upregulated. Of the stress-related genes represented in the microarray data, CDC6, GADD45A and FAS were shown to be downregulated in irradiated cells pretreated with SeM, as compared to radiation alone (Fig. 3). Additionally, the well-characterized stress response gene, ATF3 (22), which showed a significant increase in expression in response to irradiation, was observed to be downregulated when supplemented with SeM (Fig. 3). Taken together, the downregulation of these genes associated with the cellular stress response by SeM in irradiated cells may be indicative of the ability of SeM to mitigate cellular stress resulting from radiation treatment.

### SeM supplementation downregulates cell communication genes following an extended period post-irradiation

Having observed the ability of SeM to mitigate radiation-induced activation of stress-associated pathways at 6 h post-irradiation, the ability of the cells to recover was further examined with an extended recovery period. Cells exposed to a 10 cGy dose yielded negligible gene modulation when processed 16 h post-irradiation, thereby suggesting the ability of the cells to recover with time (Fig. 2B). Attempts to observe cells in the absence of SeM supplementation following 10 cGy exposure at 16 h post-irradiation also yielded negligible activation of pathways (data not shown). However, upon exposure to 20 cGy and in the presence of SeM, certain genes associated with cell communication were observed to be downregulated, when compared to the gene expression levels in irradiated cells (Fig. 2B). Particularly, the suppression of genes encoding for integrin αv, fibronectin 1 (FN1) and type IV collagen (Table V) may suggest the ability of SeM to curtail aggressive cell behavior following exposure to a higher dose of radiation.

## Discussion

Selenium is a promising cancer chemopreventive agent; its contribution to antioxidant activities is thought to be a likely mode of action. SeM has also been shown to contribute to the mitigation of radiation-induced oxidative stress (17–19). Since SeM alone is redox-inactive, the antioxidant contribution is expected to be indirect. Specifically, SeM is a nutritional source of selenium cofactor for selenoproteins and enzymes, such as glutathione peroxidase and thioredoxin reductase (13), which serve as essential antioxidant enzymes with activities which protect against oxidative stress. Indeed, the function of certain transcription factors associated with oxidative stress, including p53 (23,24), AP-1 (25) and NF-κB (25,26), are known to be oxidant-sensitive and maintained in the active state through reduction. Thus, SeM may contribute to downstream signaling via the modulation of the oxidation state of these key transcription factors during a stress response.

The signaling events associated with selenium treatment have been shown to target malignant cell lines, while sparing normal cells. For example, SeM has the ability to selectively induce apoptosis and growth arrest in cancerous, but not normal, human prostate cells (27). Likewise, gene profiling of premalignant lesions in rat mammary glands and cultured human mammary epithelial cells treated with methylseleninic acid (a potent pro-oxidant form of selenium) revealed the modulation of regulatory genes linked to cell cycle and apoptosis, properties consistent with a role in the reduction of lesion development or the death of the premalignant cells (28,29). Mechanistically, SeM treatment was shown to promote growth inhibition in human colon cancer cells through the phosphorylation of histone H3 via the activation of MAPK extracellular regulated kinase (ERK) and subsequent phosphorylation of the ribosomal S6 kinase (RSK) (30,31). Recently, the ability of selenium to alter the ECM and stroma has been suggested as being responsible for its cancer chemopreventive potential (32). A comparison of both normal and malignant prostate cell lines adapted to Se-methylselenocysteine (a source of selenium in alliums) for one month, in order to achieve steady-state selenium levels, revealed significant modulation of ECM-associated genes, such as collagens (32). As such, we observed various gene products associated with cell-cell and cell-ECM communication to be downregulated when treated with SeM and processed 16 h after 20 cGy irradiation (Table V). Although the significance of this observation is unclear, the contributions to stromal remodeling (from the alteration of ECM gene products) and cell-cell communication may suggest a possible mechanism for the chemopreventive property of selenium. As SeM suppresses radiation transformation when applied to cultures of irradiated HTori-3 cells at time periods of longer than 16 h post-irradiation (Ware *et al*, unpublished data), the effects of SeM at 16 h post-irradiation may be particularly significant for understanding the mechanism(s) by which SeM suppresses radiation transformation *in vitro*.

Our results demonstrate the ability of low-dose HZE particle radiation to elicit changes in global gene expression consistent with a stress response. The activation of genes associated with cell cycle checkpoint (CDC6), apoptosis (GADD45A and FAS) and general stress response (ATF3) is consistent with a scenario of cellular growth arrest and repair. Whereas SeM mitigates the expression of genes associated with the stress response brought about following a short period of time (6 h) post-irradiation, SeM effects on gene expression patterns at a longer period of time post-irradiation (16 h) indicate that it potentially alters cell behavior by modulating the expression of genes associated with cell adhesion.

## Figures and Tables

**Table I.** Primer sets used for real-time RT-PCR experiments.

Symbol	Forward primer (5′→3′)	Reverse primer (5′→3′)	Amplicon size (nt)
ATF3	TTTGCCATCCAGAACAAGC	CATCTTCTTCAGGGGCTACCT	121
CDC6	CCTGTTCTCCTCGTGTAAAAGC	GTGTTGCATAGGTTGTCATCG	73
FAS	GTGGACCCGCTCAGTACG	TCTAGCAACAGACGTAAGAACCA	112
GADD45A	TTGCAATATGACTTTGGAGGAA	CATCCCCCACCTTATCCAT	71
β-actin	TCGTGCGTGACATTAAGG	ACAGGTCTTTGCGGAT	258
GAPDH	AGCCACATCGCTCAGACAC	GCCCAATACGACCAAATCC	66

RT-PCR, reverse transcription polymerase chain reaction; nt, nucleotides. ATF3, cyclic adenosine monophosphate-dependent transcription factor; CDC6, cell division cycle 6; FAS, TNF receptor superfamily member 6; GADD45A, growth arrest and DNA damage-inducible protein 45A; GAPDH, glyceraldehyde 3-phosphate dehydrogenase.

**Table II.** Pathway analysis of gene induction at 10 cGy iron ion irradiation.

Symbol	Accession No.	Gene name^a^	Fold change	Pathway affected
SKP2	NM_005983	S-phase kinase-associated protein 2	2.2	Ubiquitin-mediated proteolysis
CUL2	NM_003591	Cullin 2	1.8	Ubiquitin-mediated proteolysis
UBE2G2	NM_003343	Ubiquitin-conjugating enzyme E2 G2	1.5	Ubiquitin-mediated proteolysis
DDB2	NM_000107	Damage-specific DNA binding protein 2	1.6	Ubiquitin-mediated proteolysis, p53 signaling
MDM2	NM_002392	Double minute 2	1.7	Ubiquitin-mediated proteolysis, p53 signaling
FAS	NM_000043	Fas (TNF receptor superfamily, member 6)	1.7	Ubiquitin-mediated proteolysis, p53 signaling

a Genes represented are those out of 124 unique transcripts observed to be upregulated and identified to affect the stated signaling pathways. Genes exhibiting ≥1.5-fold expression and 5% false discovery rate (FDR) were considered significant and uploaded for analysis.

**Table III.** Pathway analysis of gene induction at 20 cGy iron ion irradiation.

Symbol	Accession No.	Gene name^a^	Fold change	Pathway affected
SKP2	NM_005983	S-phase kinase-associated protein 2	2.1	Ubiquitin-mediated proteolysis
CUL5	AAB70253	Cullin 5	1.5	Ubiquitin-mediated proteolysis
WWP1	NM_007013	WW domain containing E3 ubiquitin protein ligase 1	1.5	Ubiquitin-mediated proteolysis
CUL3	AAC28621	Cullin 3	1.5	Ubiquitin-mediated proteolysis
CUL2	NM_003591	Cullin 2	1.8	Ubiquitin-mediated proteolysis
CUL4B	AAB67315	Cullin 4B	1.5	Ubiquitin-mediated proteolysis
SKP1	NM_006930	S-phase kinase-associated protein 1A	1.6	Ubiquitin-mediated proteolysis
PIAS2	AAC36704	Protein inhibitor of activated STAT, 2	1.5	Ubiquitin-mediated proteolysis
DDB2	NM_000107	Damage-specific DNA binding protein 2	1.7	p53 signaling
FAS	NM_000043	Fas (TNF receptor superfamily, member 6)	2.3	p53 signaling
TNFRSF10B	NM_003842	TNF receptor superfamily, member 10B	1.8	p53 signaling
GADD45A	NM_001924	Damage-specific DNA binding protein 2	1.6	p53 signaling, cell cycle
CDK2	NM_001798	Cyclin-dependent kinase 2	1.7	p53 signaling, cell cycle
SKP2	NM_005983	S-phase kinase-associated protein 2	2.1	p53 signaling, cell cycle
BUB3	AAC06258	Budding uninhibited by benzimidazoles 3 homolog (yeast)	1.7	Cell cycle
YWHAZ	NM_003406	Tyrosine 3-monooxygenase/tryptophan 5-monooxygenase activation protein, zeta polypeptide	1.5	Cell cycle
SKP1	NM_006930	S-phase kinase-associated protein 1A	1.6	Cell cycle
RIPK1	NM_003804	Receptor (TNFRSF)-interacting serine-threonine kinase 1	1.7	Apoptosis
FAS	NM_000043	Fas (TNF receptor superfamily, member 6)	2.3	Apoptosis
BCL2L1	NM_001191	BCL2-like 1	1.5	Apoptosis
TNFRSF10B	NM_003842	TNF receptor superfamily, member 10B	1.8	Apoptosis
CFLAR	NM_003879	Tumor necrosis factor receptor superfamily, member 10B	1.5	Apoptosis
SKP2	NM_005983	S-phase kinase-associated protein 2	2.1	Small cell lung cancer
CDK2	NM_001798	Cyclin-dependent kinase 2	1.7	Small cell lung cancer
BCL2L1	NM_001191	BCL2-like 1	1.5	Small cell lung cancer
TRAF3	AAA56753	TNF receptor-associated factor 3	1.5	Small cell lung cancer
PIAS2	AAC36704	Protein inhibitor of activated Stat, 2	1.5	Small cell lung cancer

a Genes represented are those out of 285 unique transcripts observed to be upregulated and identified to affect the stated signaling pathways.

**Table IV.** Pathway analysis of gene modulation after SeM treatment in sham-irradiated cells and in cells exposed to a 10 cGy dose of radiation.

Symbol	Accession No.	Gene name^a^	Fold change	Pathway affected
Sham-irradiated				
CCND3	NM_001760	Cyclin D3	1.5	Cell cycle
CDC20	NM_001255	Cdc20 cell division cycle 20 homolog (S. cerevisiae)	1.5	Cell cycle
MAD1L1	NM_001013836	Mad1 mitotic arrest deficient-like 1 (yeast)	2.0	Cell cycle
CCND1	NM_053056	Cyclin D1	1.5	Cell cycle
BUB1	AAB97855	Budding uninhibited by benzimidazoles 1 homolog	1.5	Cell cycle
PGM3	NM_015599	Phosphoglucomutase 3	1.9	Pentose phosphate
GPI	NM_000175	Glucose phosphate isomerase	1.7	Pentose phosphate
PGD	NM_002631	Phosphogluconate dehydrogenase	1.7	Pentose phosphate
10 cGy^b^				
PGD	NM_002631	Phosphogluconate dehydrogenase	1.5	Pentose phosphate, glycolysis/gluconeogenesis
PGM3	NM_015599	Phosphoglucomutase 3	1.6	Pentose phosphate glycolysis/gluconeogenesis
GPI	NM_000175	Glucose phosphate isomerase	1.7	Pentose phosphate glycolysis/gluconeogenesis
ENO1	NM_001428	Enolase 1	1.9	Glycolysis/gluconeogenesis
MET	NM_000245	Met proto-oncogene	0.6	Adherens junction
IQGAP1	NM_003870	IQ motif containing GTPase activating protein 1	0.6	Adherens junction

a Genes represented are those out of 97 unique transcripts observed to be upregulated and identified to affect the stated signaling pathways for sham-irradiation with SeM treatment.

b 6 h post-irradiation. For 10 cGy irradiation and SeM treatment, 61 unique transcripts were modulated. SeM, L-selenomethionine.

**Table V.** Pathway analysis of downregulated genes in response to SeM treatment with cells harvested at 16 h post-irradiation.**

Symbol	Accession No.	Gene name^a^	Fold change	Pathway affected
10 cGy				
No identifable pathways				
20 cGy				
MET	NM_000245	Met proto-oncogene	0.7	Focal adhesion
VCL	NM_003373	Vinculin	0.6	Focal adhesion
ITGAV	NM_002210	Integrin, alpha V	0.6	Focal adhesion, extracellular matrix receptor interaction
LAMB1	NM_002291	Laminin, beta 1	0.5	Focal adhesion, cell communication, extracellular matrix receptor interaction
TNC	NM_002160	Tenascin C	0.3	Focal adhesion, cell communication, extracellular matrix receptor interaction
THBS1	NM_003246	Thrombospondin 1	0.6	Focal adhesion, cell communication, extracellular matrix receptor interaction
COL4A2	NM_001846	Collagen, type IV, alpha 2	0.5	Focal adhesion, cell communication, extracellular matrix receptor interaction
COL4A1	NM_001845	Collagen, type IV, alpha 1	0.5	Focal adhesion, cell communication, extracellular matrix receptor interaction
FN1	NM_002026	Fibronectin 1	0.5	Focal adhesion, cell communication, extracellular matrix receptor interaction
LAMC1	NM_002293	Laminin, gamma 1	0.5	Focal adhesion, cell communication, extracellular matrix receptor interaction
DSC3	NM_001941	Desmocollin 3	0.6	Cell communication
DSG2	AAH99655	Desmoglein 2	0.6	Cell communication
CDH2	NM_001792	Cadherin 2, type 1, n-cadherin	0.6	Cell adhesion molecules
PTPRF	NM_002840	Protein tyrosine phosphatase, receptor type F	0.7	Cell adhesion molecules
NEO1	NM_002499	Neogenin homolog 1	0.7	Cell adhesion molecules
GLG1	NM_012201	Golgi apparatus protein 1	0.6	Cell adhesion molecules
VCAN	NM_004385	Chondroitin sulfate proteoglycan 2 (Versican)	0.4	Cell adhesion molecules
NCAM1	NM_000615	Neural cell adhesion molecule 1	0.6	Cell adhesion molecules
ITGAV	NM_002210	Integrin, alpha V	0.6	Cell adhesion molecules, small cell lung cancer
COL4A1	NM_001845	Collagen, type IV, alpha 1	0.5	Small cell lung cancer
FN1	NM_001846	Fibronectin 1	0.5	Small cell lung cancer
COL4A2	NM_002026	Collagen, type IV, alpha 2	0.5	Small cell lung cancer
LAMB1	NM_002291	Laminin, beta 1	0.5	Small cell lung cancer, Prion disease
LAMC1	NM_002293	Laminin, gamma 1	0.6	Small cell lung cancer, Prion disease
HSPA5	NM_005347	Heat shock 70 kDa protein 5	0.5	Prion disease
PTPRF	NM_002840	Protein tyrosine phosphatase, receptor type F	0.6	Adherens junction
INSR	NM_000208	Insulin receptor	0.6	Adherens junction
MET	NM_000245	Met proto-oncogene	0.7	Adherens junction
VCL	NM_003373	Vinculin	0.6	Adherens junction

a With SeM treatment of the 10 cGy irradiated cells, 15 genes were observed to be downregulated, but no significant effects on signaling pathways were observed. In contrast, with SeM treatment of the 20 cGy irradiated cells, genes are represented out of 123 unique transcripts observed to be downregulated and identified to affect the stated signaling pathways. SeM, L-selenomethionine.
